# Investigating the Effects of NaCl on the Formation of AFs from Gluten in Cooked Wheat Noodles

**DOI:** 10.3390/ijms24129907

**Published:** 2023-06-08

**Authors:** Ying Liang, Jiayang Song, Jiayi Wang, Hao Liu, Xingquan Wu, Baoshan He, Xia Zhang, Jinshui Wang

**Affiliations:** 1 College of Biological Engineering, Henan University of Technology, Zhengzhou 450001, China; yliang@haut.edu.cn (Y.L.); sjy035070@gmail.com (J.S.); wjy0536@gmail.com (J.W.); liuhao0051@gmail.com (H.L.); lying2048@gmail.com (X.W.); flowersas@163.com (X.Z.); 2 College of Food Science and Engineering, Henan University of Technology, Zhengzhou 450001, China; hebaoshan@126.com

**Keywords:** wheat noodle, gluten, amyloid fibrils, NaCl

## Abstract

To clarify the effect of NaCl concentration (0–2.0%) on the formation of amyloid fibrils (AFs) in cooked wheat noodles, the morphology, surface hydrophobicity, secondary structure, molecular weight distribution, microstructure, and crystal structure of AFs were investigated in this paper. Fluorescence data and Congo red stain images confirmed the presence of AFs and revealed that the 0.4% NaCl concentration promoted the production of AFs. The surface hydrophobicity results showed that the hydrophobicity of AFs increased significantly from 3942.05 to 6117.57 when the salt concentration increased from 0 to 0.4%, indicating that hydrophobic interactions were critical for the formation of AFs. Size exclusion chromatography combined with gel electrophoresis plots showed that the effect of NaCl on the molecular weight of AFs was small and mainly distributed in the range of 5–7.1 KDa (equivalent to 40–56 amino acid residues). X-ray diffraction and AFM images showed that the 0.4% NaCl concentration promoted the formation and longitudinal growth of AFs, while higher NaCl concentrations inhibited the formation and expansion of AFs. This study contributes to the understanding of the mechanism of AF formation in wheat flour processing and provides new insight into wheat gluten aggregation behavior.

## 1. Introduction

Amyloid fibrils (AFs) are a kind of protein aggregation rich in cross-β-folding structures obtained by special protein processing [1,2]. AFs have been extensively studied in the food sector, such as β-lactoglobulin [3,4], soy proteins [5,6], and egg proteins [7], which are able to self-assemble to form protofibrils by hydrophobic interactions, electrostatic forces, hydrogen bonding, and van der Waals forces under the conditions of low pH, low ionic strength, and high or prolonged heating [8]. In addition, AFs have properties such as high Young’s modulus, high tensile strength, high hydrophobicity, antioxidant and antibacterial activity, and good stability and interface properties [9]. In recent years, the fibrillation of food proteins has been considered as a strategy to improve the functional properties of foods [7], which could meet the needs of the development of the functional food industry [10,11]. Wheat protein is a food protein that makes up a large percentage of the daily diet [12], but research on its fibrillation is still limited. 

Gluten is a protein unique to wheat flour and its unique viscoelasticity plays an irreplaceable role in flour products [13]. Gluten proteins rich in high levels of glutamine and hydrophobic amino acids have been shown to be associated with the formation of AFs [14,15]. Heat processing is indispensable for most wheat flour products. A series of physical and chemical reactions occur during the hot processing of wheat flour products, which have a significant influence on the formation of the gluten network structure and the final properties of the product [13,16]. Monge-Morera, M et al. found that gluten proteins can promote amyloid fibrillar protein formation during heating [17]. NaCl is widely used in the processing of pasta products. NaCl has been shown to improve noodle texture [18], improve the rheological properties of dough [19], and also have significant effects on the formation and polymerization of gluten networks [20]. Li et al. found that NaCl was able to induce the fibrillation of the gluten network structure in the noodle system [21]. Salt ions can also influence protein folding and unfold through ionic interactions [22]. Although there have been some studies on gluten fibrillation [17,23], the effect of NaCl on the formation of AFs during the thermal processing of wheat flour products under food processing conditions has not been studied.

Based on the above background, the aim of this study was to systematically investigate the effect of NaCl on the formation of AFs in thermally processed pasta products. Therefore, in this study, we used the thioflavin T fluorescence method and Congo red staining to detect and identify AFs. Then, the particle size, surface hydrophobicity, and molecular weight distribution of AFs were comparatively evaluated. Finally, the secondary structure, molecular chain morphology, and crystal structure of amyloid fibrils were analyzed by Fourier infrared spectroscopy, atomic force microscopy, and X-ray diffraction. This study will provide a new solution to regulate the content of AFs in wheat flour products and has important guiding significance for the application of AFs in food.

## 2. Results and Discussion

### 2.1. Analysis of ThT Fluorescence

Thioflavin T binds specifically to cross-beta structures and the framework of the beta-sheet, and its fluorescence intensity can characterize changes in the content of AFs [24]. The fluorescence intensity of the extracted AFs after heating with different NaCl concentrations (0–2%) is shown in Table 1 below. The fluorescence intensity of AFs increased and then decreased with an increasing salt concentration, reaching a peak at a 0.4% NaCl concentration. The fluorescence intensity of the protein samples increased, indicating that the addition of an appropriate concentration of NaCl could promote the formation of AFs. The literature reports that starch and protein mixing does not affect polymerization kinetics during heating [25,26], which greatly reduces the effect on fibrin aggregation. The addition of a low concentration of NaCl can reduce the activation enthalpy during fibril nucleation, produce more polypeptides, and promote the formation of AFs [27]. In conclusion, a NaCl concentration of 0.4% promoted protein fibrillation, while higher NaCl concentrations had a significant inhibitory effect on fibril growth.

### 2.2. Congo Red Polarized Light Microscope Observation

**Figure 1:**
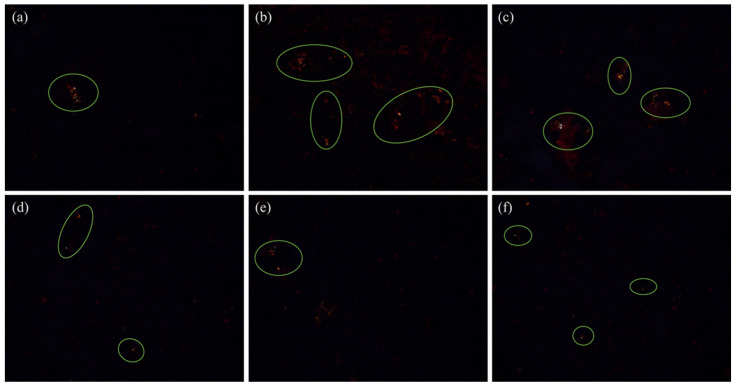
Congo red staining polarizing microscope observation. The green coils represent the green refraction phenomenon of β-sheets in AFs specifically binding with Congo red. Images (**a**–**f**) represent the results of Congo red staining of amyloid fibrin at 0%–2.0% NaCl concentration in that order.

Congo red is an azo dye with a selective affinity for AFs. The hydroxyl groups on polysaccharide molecules in amyloid can bind to amino groups on dye molecules, thus attaching to AFs and exhibiting the typical green birefringence [17]. The results of the Congo red staining of AFs at different salt concentrations are shown in Figure 1 below. Combined with the fluorescence data, the fluorescence intensity at a 0.4% NaCl concentration was consistent with the distribution of AFs in Figure 1b and further confirmed the presence of AFs.

### 2.3. Particle Size Analysis

The particle size distribution of AFs formed under different NaCl concentrations was studied. From Table 1, it can be seen that the average particle size of AFs is the largest at a 0.4% salt concentration, with a general trend of increasing and then decreasing. This may be due to the fact that a low NaCl concentration exposes more hydrophobic groups within protein molecules and enhances intermolecular hydrophobic interactions, prompting AFs to aggregate into larger aggregates, resulting in a larger particle size distribution. In contrast, under (0.4–2.0%) NaCl concentration conditions, the surface charge of protein molecules increased with an increasing salt concentration, resulting in the mutual repulsion of protein molecules and enhanced interaction with water molecules, thus reducing the degree of aggregation between protein molecules [28].

### 2.4. H0 Analysis

H0 determines the degree of exposure of hydrophobic groups on the protein surface, which is a sign of a change in the tertiary structure of the protein [29]. ANS is widely used as a fluorescent dye preferentially bound to hydrophobic groups, and it is widely used to detect the exposure of hydrophobic groups during fibrillation [11]. Table 1 shows the changes in AF H0 under different NaCl concentrations. As shown in Table 1, the H0 of the AF samples was maximum at a 0.4% salt concentration and then gradually decreased with an increasing salt concentration. Compared to the control, the H0 of AFs at a 0.4% salt concentration increased significantly from 3942.05 to 6117.57. It is possible that the protein structure under low ionic strength heating is disrupted and hydrolyzed into lower-molecular-weight peptide segments with more hydrophobic groups exposed inside, which then form AFs by self-assembly. The exposure of hydrophobic groups in proteins increases the contact area of protein molecules, thus contributing to the formation of AFs [30]. The results are consistent with those of the polarizing microscope and fluorescence detection. It is shown that protein hydrophobic groups are involved in the self-assembly of AFs [31]. This is due to the hydrophobic interactions existing between hydrophobic groups that facilitate the formation of β-sheets, while exposed aromatic amino acids are also involved in the formation of fibrils [32]. Combined with the particle size data, the hydrophobic interactions and electrostatic repulsion between gluten molecules reached a balanced state at a low NaCl concentration, which facilitated fibril formation and elongation. However, with the increase in NaCl concentration, the surface charge of protein molecules increased, the electrostatic repulsion was enhanced, and the balance of hydrophobic interactions and electrostatic forces between protein molecules was disrupted, which was unfavorable to the growth of cellulose.

### 2.5. Sodium Dodecyl Sulfate-Polyacrylamide Gel Electrophoresis (SDS-PAGE)

**Figure 2:**
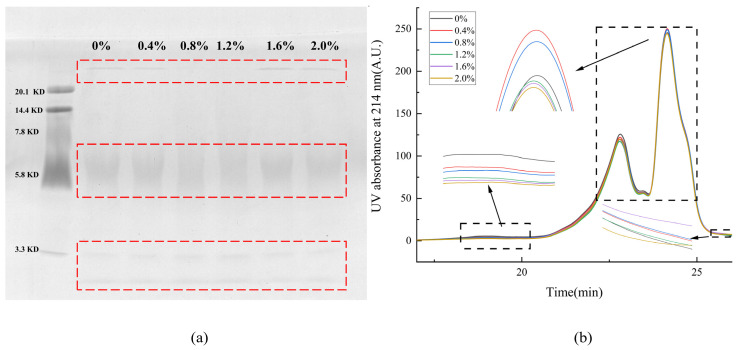
Effect of NaCl on the molecular weight distribution of AFs. (**a**) Electrophoretic map of AFs at 0–2.0% NaCl concentration. The red coils indicate different molecular weight ranges of proteins. (**b**) Size exclusion map of AFs at 0–2.0% NaCl concentration. The black frames indicate the different molecular weight distributions of the proteins.

In gel electrophoresis, the migration rate of protein subunits is negatively correlated with the relative molecular weight of protein subunits, which means that the higher the relative molecular weight, the slower the mobility. In order to determine the molecular weight distribution of protein in AFs, the protein samples at different NaCl concentrations were analyzed by SDS-PAGE. The molecular weight distribution of AFs is shown in Figure 2 below. NaCl had no significant effect on the molecular weight distribution of AFs. According to the low-molecular-weight protein maker calibration, the molecular weights of the proteins in the samples were mainly distributed in three regions of less than 3.3 KDa, 5–7.1 KDa, and greater than 20.1 KDa. Through analytical calculations, the molecular weight distribution of AFs was equivalent to more than 158 amino acid residues at >20.1 KDa, 40–56 amino acid residues at a molecular weight distribution of 5–7.1 KDa, and less than 26 amino acid residues at molecular weight below 3.3 KDa. Proteins with molecular weights below 3.3 KDa are probably products of proteinase K and trypsin inactivation.

### 2.6. Changes in Molecular Weight Distribution 

The solubility of proteins in an SDS solution is determined by SE-HPLC, which can be used to characterize the degree of the cross-linking of proteins [33]. Figure 2 shows the SE-HPLC spectra of AF extracts at different salt concentrations. The elution spectra showed that the AF extracts were composed of three components with elution times of 18.2–20.3 min (component 1), 20.7–25.4 min (component 2), and 25.4–26.5 min (component 3), which were consistent with the results of SDS-PAGE. Combined with the fluorescence data, it can be seen that the peak area of the elution time is consistent with the change in fluorescence intensity when the elution time is 23–25.4 min. This experiment mainly focused on the AF elution at 23–25.4 min.

### 2.7. Fourier Infrared Spectrum Analysis

FTIR is a common technique for the determination of protein conformation [34]. Most information about the secondary structure of proteins can be obtained from the amide I region of the spectrum (1700–1600 cm^−1^) [35]. In order to further explore the AF structure extracted from noodle freeze-dried powder under different NaCl concentrations, the infrared spectra of the samples were measured. In the FTIR spectrum, the amide I band (1600–1700 cm^−1^) is very sensitive to the secondary structural changes of the protein during denaturation and aggregation. The structure distribution of AFs in the amide I zone under different NaCl concentrations is shown in Table 2 below. Deconvolution spectra show four bands in the amide I region, representing β-folding (1600–1635 cm^−1^), random curling (1635–1644 cm^−1^), α-helix (1645–1665 cm^−1^), and β-turns (1666–1700 cm^−1^). As seen in Table 2, compared to the control group, the β-folded content increased significantly at a 0.4% salt concentration, reached a peak, and then gradually decreased with an increasing salt concentration. At a 0.4% salt concentration, the β-turn and random coil structures of the AFs tended to shift toward β-folded and α-helix. This also shows that the salt concentration of 0.4% is not only favorable to promote fibronectin formation but is also the best condition to form structures containing a high level of α-helix. In the secondary structure of AFs at a 0.4–2.0% salt concentration, the contents of β-folded and random curling structures decreased gradually with the increase in salt concentration, while the contents of α-spiral structures remained essentially unchanged, and the contents of β-turns increased. This indicated that a high salt concentration inhibited β-turns and the transition of a random curling structure to β-folding and was not conducive to the growth of AFs. This is consistent with the previous findings that an appropriate salt concentration can change the content of the secondary structure and promote the formation of fibrils [36], while a high salt concentration will increase the irregular curling structure and hinder the formation of fibrils [22].

### 2.8. X-ray Diffraction Analysis

**Figure 3:**
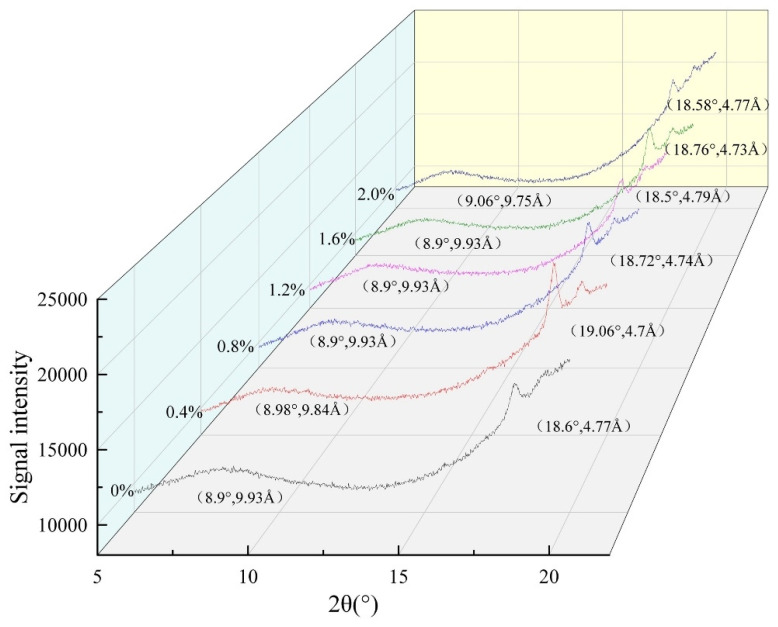
X-ray diffraction profiles of AFs at different NaCl concentrations. 0–2.0% represents the range of NaCl additions in AFs.

XRD was used to study the crystal structure of AFs perpendicular to the fiber axis under different salt concentrations, as shown in Figure 3 below. XRD is a technique that uses the diffraction effect of X-rays to analyze the structure of crystalline materials. Each crystalline material has its own specific crystal structure, including lattice type, crystal plane spacing, and other parameters, and AFs contain a typical crossed β-sheet structure. The AFs have a typical XRD pattern with a meridional reflection of about 4.8 Å, corresponding to the β-sheet spacing, while the independent equatorial reflection of the protein is about 6–12 Å, corresponding to the distance between stacked β-sheets (spacing is generally 10 Å) [37,38]. As can be seen from the diffraction pattern in Figure 3, with the increase in salt concentration, the chain spacing of β-sheets in AFs is between 4.7 Å and 4.8 Å, and the layer spacing is about 9.9 Å. This is consistent with the study of the crystal spacing of AFs in the literature. It also indicated that the addition of NaCl had little effect on the β-sheets in AFs, and the structure of the β-sheets formed was stable.

### 2.9. AFM Analysis

**Figure 4:**
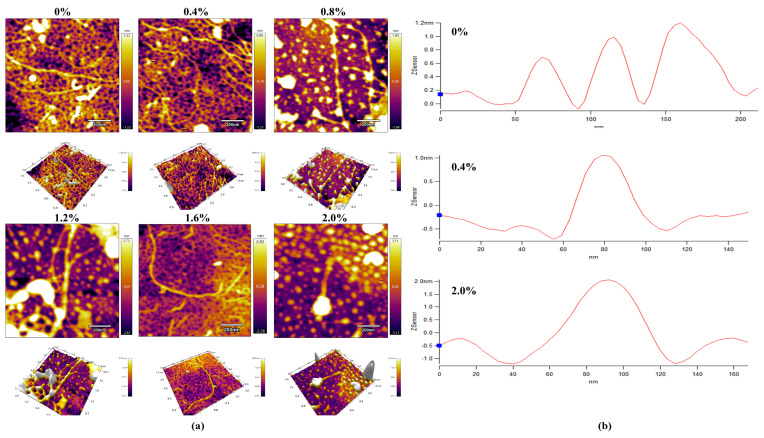
Contour length (**a**) and maximum height distribution (**b**) of AFs under atomic force microscopy. 0–2.0% represents the range of NaCl additions in AFs.

In order to study the effect of NaCl concentration on the morphology of AFs, high-resolution images of all samples at the nanoscale were collected. Figure 4 shows the length of the profile (a) and the maximum height distribution (b) of the AFs under different NaCl treatments. The results of the AF images showed that the height of AFs increased with an increasing salt concentration (Figure 4b). The AFs added with a low concentration of NaCl (Figure 4. 0.4%) formed the largest number of long fibrils with branches, which was consistent with the results of fluorescence. However, when the NaCl concentration was higher (0.4–2.0%), the elongation of the fibrils was hindered, and more short fibrils formed, accompanied by the formation of small aggregates. The reason could be that a higher NaCl concentration breaks the electrostatic equilibrium, and enhanced electrostatic repulsion inhibits the elongation of the fibrils. In conclusion, the morphology of AFs is different under different NaCl concentrations. A low NaCl concentration promotes the formation and longitudinal growth of AFs, while a high NaCl concentration inhibits the elongation of fibrils and the formation of small oligomers. It was found that there is a dynamic balance between monomers, oligomers, protofibrils, mature protofibrils, and amorphous aggregates, where hydrophobic interactions are an important factor in determining the type of aggregation [39].

## 3. Materials and Methods

### 3.1. Materials

Family Banquet Wheat Flour, purchased from Cofco International (Beijing, China) Co., Ltd. SDS-PAGE Gel preparation kit, purchased from Beijing Solaibao Technology Co., Ltd. (Beijing, China). Low-molecular-weight protein Marker (3.3–20.1 KDa), purchased from Beijing Solaibao Technology Co., Ltd. Thioflavin T (2390-54-7, 97% purity), purchased from Shanghai Maclin Biochemical Technology Co., Ltd. (Shanghai, China) Congo red Indicator, purchased from Tianjin Kemi Ou Chemical Reagent Co., Ltd. (Tianjin, China). 8-aniline-1 naphthalene sulfonic acid (ANS, purity ≥ 96%), purchased from Shanghai Maclin Biochemical Technology Co., Ltd. All chemical reagents used were of analytical grade.

### 3.2. Preparation of Noodles

Referring to the noodle preparation method of Liang et al. [40]. A total of 100 g of wheat flour was mixed with water at 1:0.4 (*w*/*w*), and NaCl was added at x% (x = 0.4, 0.8, 1.2, 1.6, 2.0) (*w*/*w*). The group without NaCl was the control group.

### 3.3. Extraction of AFs

A slightly modified version of the research method of Lambrecht et al. [23] was used. A total of 1.5 g freeze-dried noodle powder and 1 mg alpha-amylase (2900 U) were blended with 10 mL 0.02% sodium azide solution, shaken at 37 °C for 24 h, and centrifuged (8000 rpm, 10 min), and the supernatant was removed. To the precipitate, 1 mg of proteinase K (26 U) solution and 10 mg of trypsin (2500 U) were added, mixed with 10 mL of sodium azide solution, and shaken for 48 h. Next, the supernatant was removed with boiling water bath for 5 min and centrifugation (8000 rpm, 10 min). The precipitate was added to 8 mL (0.05 M, pH 7.0) phosphate buffer, shaken for 16 h at 150 rpm at 25 °C, and centrifuged, and the supernatant was retained. The supernatant was freeze-dried, ground, passed through 100 mesh, and set aside in a desiccator.

### 3.4. Thioflavin T Fluorescence (ThT)

Referring to Loveday et al. with slight modifications [3], the 3.0 mM ThT stock solution prepared by phosphate-NaCl buffer (0.01 M phosphate buffer and 150 mM NaCl, pH 7.0) was filtered through a 0.22 μm filter membrane (PES, Jinteng, Zhoushan, China) and stored in brown glass bottles at 4 °C. The working solution was prepared by diluting the stock solution 50 times in the phosphate-NaCl buffer (the final ThT concentration was 60 μM). During the experiment, 50 μL sample was added to 3 mL working solution. The mixture was briefly rotated and held at room temperature for 1 min, and then fluorescence was measured (Cary Eclipse, Agilent, Penang, Malaysia). The excitation wavelength was 440 nm, and the emission wavelength was 482 nm.

### 3.5. Congo Red Staining Observation 

AFs exhibit typical green birefringence when stained with Congo red. A total of 10 μL of concentrated protein solution was placed on a slide, 20–30 μL of staining solution (80% ethanol solution, excess NaCl, excess Congo red, appropriate amount of 0.05% sodium azide, stirred and filtered) was applied to the dried protein samples, and the excess solution was blotted off with filter paper. Care was taken not to touch the sample and to allow the dyed sample to dry at room temperature. The stained samples were then examined with a polarized light microscope (50ipol, Nikon Stock Corporation, Tokyo, Japan). Yellow/green birefringence tests are considered positive for the presence of amyloid [41].

### 3.6. Particle Size Analysis

The particle size was measured according to a known procedure [16]. The AF samples were diluted to 1 mg/mL. The particle size distribution and D50 values of the samples were determined using a laser particle size analyzer (Mastersizer-3000. Malvern Instruments Ltd., Malvern, UK) at 14% shading. All measurements were performed at 25 °C in triplicate.

### 3.7. Surface Hydrophobicity (H0)

The surface hydrophobicity of the proteins was determined using 1-aniline-8-naphthalenesulfonate (ANS) in duplicate [23]. Samples were diluted with 0.01 M sodium phosphate buffer (pH 7.0) to obtain protein concentrations of 0.05–0.50 mg/mL. An aliquot (200 μL) of the diluted sample was then transferred to a black 96-well plate (Greiner Bio-One) with 10 μL of ANS solution (8.0 mM). Fluorescence analysis was performed using a multifunctional microplate fluorescence reader (Spark, Tecan, Männedorf, Switzerland) with excitation and emission wavelengths of 390 and 480 nm. The relative fluorescence intensity was calculated as the fluorescence intensity of the protein-ANS mixture minus the fluorescence intensity of the ANS solution. The slope of the plot of the relative fluorescence intensity of each sample as a function of protein concentration represents the surface hydrophobicity.

### 3.8. Sodium Dodecyl Sulfate-Polyacrylamide Gel Electrophoresis (SDS-PAGE)

The molecular weight distribution of AFs was determined by SDS-PAGE assay with reference to the method of Laemmli [42]. A 20% separation gel and a 5% concentration gel were used. The freeze-dried sample was mixed with Laemmli buffer (pH 6.8) solution (consisting of 62.5 mmol/L Tris-HCl, 10% (*v*/*v*) glycerol, 5% (*v*/*v*) mercaptoethanol, 2% (*w*/*v*) SDS, and 0.25% (*w*/*v*) bromophenol blue) to a concentration of 40 mg/mL. Then, it was centrifuged at 10,000 rpm for 5 min at room temperature and incubated at 100 °C for 5 min. Ten-microliter equivalents of each sample were loaded into the lane. SDS-PAGE (DYY-7C Electrophoresis apparatus) used 150 V. The gel was stained with Coomassie bright blue R-250 (Shanghai Blue Season Biology, Shanghai, China).

### 3.9. Size Exclusion High-Performance Liquid Chromatography (SE-HPLC)

The molecular weight distribution of AFs was detected with the LC system referring to the method of Lambrecht et al. (LC-2010, Shimadzu, Kyoto, Japan) [23]. The 1.0 mg protein sample was dissolved in 1 mL 0.050 M sodium phosphate buffer (pH 6.8) containing 2.0% (*w*/*v*) SDS and shaken (60 min, room temperature). After centrifugation (10,000 rpm, 10 min) and filtration (0.45 μm, PES, microporous, Tianjin Jinteng, Tianjin, China), the protein sample solution (20 μL) was loaded onto a BioSep SECS4000 column (5 μm, 300 mm × 7.8 mm, Phenomenex, Torrance, CA, USA) at a rate of 1 mL/min for analysis. The elution solvent (acetonitrile/water (1:1, *v*/*v*) containing 0.1% (*v*/*v*) trifluoroacetic acid) was used at a flow rate of 1 mL/min, and the UV detector intensity was 214 nm [43].

### 3.10. Fourier Transform Infrared Spectrum (FTIR)

Fourier transform infrared (FT-IR) spectroscopy with a Tensor II spectrometer (Bruker, Mannheim, Germany) was used to obtain FTIR spectra of AFs in noodle flour [44]. A total of 5 mg of powder sample mixed with 500 mg of potassium bromide was ground well and pressed into a thin tablet. Fourier infrared spectrometer was used to scan the whole band (400–4000 cm^−1^) 64 times. Deconvolution and second-order fitting of 1600–1700 cm^−1^ amide I were performed using Peak Fit 4.0 software.

### 3.11. X-ray Diffraction (XRD)

The crystal structure of AFs perpendicular to the fiber axis was studied by XRD [17]. The diffraction pattern conditions for AFs were 2θ: 5–40° at a rate of 5°/min. Analysis and display were performed using MDI Jade 6 and origin 2021 software. 

### 3.12. Atomic Force Microscope (AFM)

The information on the molecular chain morphology of AFs was observed with AFM (MFP-3D Infinity, Oxford, America) in AC mode [45]. Protein samples were dissolved in 0.05 M acetic acid solution and then diluted with a gradient of acetic acid solution to obtain a concentration of approximately 0.01 µg/mL protein solution. A total of 10 μL of the protein solution was taken as a drop on a fresh mica sheet, air-dried, and then observed in AFM.

### 3.13. Statistical Analysis

All measurements were made in triplicate and averaged. IBM SPSS 26 statistical software was used to analyze the data. Univariate analysis of variance (ANOVA) was used to analyze whether there were significant differences among multivariate variables (*p* < 0.05). Data were plotted using Origin 2021.

## 4. Conclusions

In this research, the fluorescence intensity, morphology, particle size, and surface hydrophobicity of AFs in cooked wheat noodles at different NaCl concentrations were measured, and the changes in AFs were further elucidated in terms of protein molecular weight, molecular chain morphology, and crystal structure. The contribution of a low concentration of NaCl to AFs was increasing the yield and length of AFs. The increase in the fluorescence intensity and β-sheet structure content of AFs with the addition of 0.4% NaCl indicates that a low NaCl concentration enhances hydrophobic interactions between proteins, which promotes the production of AFs and facilitates the unidirectional ordered extension of fibrils. This was also the optimal condition that led to the production of fibrils with the highest α-helix content. The strongest hydrophobic index of the AFs formed at a 0.4% salt concentration was 6117.57, indicating that hydrophobic interactions are the dominant force in protein self-assembly. The chain spacing of β-sheets in AFs was found to be between 4.7 and 4.8 Å, and the layer spacing was around 9.9 Å in XRD patterns, which is consistent with known literature reports. The results of AFM images showed that the height of the AFs gradually increased with the addition of NaCl. The 0.4% NaCl concentration promoted the lateral growth of AFs, forming more protein-length fibers, and then had an inhibitory effect on the elongation of AFs as the salt concentration increased. In conclusion, the addition of NaCl can affect the interaction between proteins, thus affecting the formation and growth of AFs. Hydrophobic interaction is the key to the formation of AFs. In addition, the structure of AFs is more stable, which is conducive to improving the functional properties of gluten protein and expanding its application range. This study systematically discussed the effect of NaCl concentration on AF formation in wheat flour products, which provided a new idea for the application of AFs in the food field.

## Figures and Tables

**Table 1:** Effects of NaCl on the content of AFs, particle size, and surface hydrophobicity.

NaCl Concentration (%)	Fluorescence Intensity	Grain Size (d/nm)	Hydrophobicity (mL/mg)
0	7093 ± 107.16 ^b^	313.93 ± 8.023 ^c^	3942.05 ± 84.52 ^d^
0.4	7332 ± 100.43 ^a^	373.40 ± 13.00 ^a^	6117.57 ± 198.07 ^a^
0.8	6634 ± 105.33 ^c^	333.87 ± 17.01 ^bc^	5585.34 ± 184.12 ^b^
1.2	6288 ± 77.57 ^d^	342.93 ± 12.72 ^b^	5191.56 ± 145.00 ^c^
1.6	6158 ± 80.02 ^d^	329.73 ± 6.568 ^bc^	3633.17 ± 295.20 ^de^
2.0	5630 ± 62.05 ^e^	324.40 ± 5.895 ^bc^	3282.54 ± 182.42 ^e^

Means with small superscript letters within the same column are significantly different at *p* < 0.05.

**Table 2:** Effects of NaCl on the secondary structure of AFs.

NaCl Concentration (%)	β-Sheet (%)	Unordered (%)	α-Helix (%)	β-Turn (%)
0.0	8.4 ± 3.3 ^bc^	13 ± 0.38 ^bc^	26 ± 0.40 ^b^	52 ± 0.18 ^ab^
0.4	11 ± 0.1 ^a^	12 ± 0.82 ^c^	37 ± 1.3 ^a^	40 ± 2.1 ^c^
0.8	10 ± 1.5 ^ab^	14 ± 0.64 ^ab^	26 ± 0.24 ^b^	49 ± 1.9 ^b^
1.2	8.6 ± 1.4 ^abc^	16 ± 0.39 ^a^	26 ± 0.81 ^b^	49 ± 0.23 ^b^
1.6	8.1 ± 0.58 ^bc^	14 ± 0.17 ^ab^	26 ± 0.21 ^b^	52 ± 0.54 ^ab^
2.0	6.9 ± 0.65 ^c^	13 ± 1.2 ^bc^	26 ± 0.18 ^b^	54 ± 0.38 ^a^

Means with small superscript letters within the same column are significantly different at *p* < 0.05.

## Data Availability

Data will be made available on request.
